# Sex hormones and gene expression signatures in peripheral blood from postmenopausal women - the NOWAC postgenome study

**DOI:** 10.1186/1755-8794-4-29

**Published:** 2011-03-31

**Authors:** Marit Waaseth, Karina S Olsen, Charlotta Rylander, Eiliv Lund, Vanessa Dumeaux

**Affiliations:** 1 Department of Community Medicine, University of Tromsø, Tromsø, Norway; 2 Norwegian Institute for Air Research, Tromsø, Norway

## Abstract

**Background:**

Postmenopausal hormone therapy (HT) influences endogenous hormone concentrations and increases the risk of breast cancer. Gene expression profiling may reveal the mechanisms behind this relationship.

Our objective was to explore potential associations between sex hormones and gene expression in whole blood from a population-based, random sample of postmenopausal women

**Methods:**

Gene expression, as measured by the Applied Biosystems microarray platform, was compared between hormone therapy (HT) users and non-users and between high and low hormone plasma concentrations using both gene-wise analysis and gene set analysis. Gene sets found to be associated with HT use were further analysed for enrichment in functional clusters and network predictions. The gene expression matrix included 285 samples and 16185 probes and was adjusted for significant technical variables.

**Results:**

Gene-wise analysis revealed several genes significantly associated with different types of HT use. The functional cluster analyses provided limited information on these genes. Gene set analysis revealed 22 gene sets that were enriched between high and low estradiol concentration (HT-users excluded). Among these were seven oestrogen related gene sets, including our gene list associated with systemic estradiol use, which thereby represents a novel oestrogen signature. Seven gene sets were related to immune response. Among the 15 gene sets enriched for progesterone, 11 overlapped with estradiol. No significant gene expression patterns were found for testosterone, follicle stimulating hormone (FSH) or sex hormone binding globulin (SHBG).

**Conclusions:**

Distinct gene expression patterns associated with sex hormones are detectable in a random group of postmenopausal women, as demonstrated by the finding of a novel oestrogen signature.

## Background

Previous reports have shown that there is an association between plasma/serum concentrations of endogenous sex hormones and the risk of breast cancer among postmenopausal women [1-3]. The Women's Health Initiative [4] and large observational studies [5,6] have also shown that use of postmenopausal hormone therapy (HT) increases the risk of breast cancer. Exogenous hormones have an influence on endogenous hormone concentrations. Systemically administered HT containing estradiol (E_2_) suppresses plasma concentrations of follicle stimulating hormone (FSH) and increases E_2 _and sex hormone binding globulin (SHBG) concentrations [7-9]. Tibolone use suppresses both FSH and SHBG concentrations in blood and increases free testosterone (T) because of lower SHBG levels [10,11].

Blood is a fluid connective tissue that interacts with all other human tissues, and peripheral blood cells have been found to reflect system wide biology [12,13]. Being easily accessible, peripheral blood can be an excellent surrogate tissue for exploring the effects of environmental exposure on gene expression in large epidemiological studies. Microarray analysis of the blood transcriptome may shed light on the etiologic pathways connecting environmental exposure and disease[13], and gene expression signatures are hypothesised to become important diagnostic tools or prognostic biomarkers [14].

Except for previous research in the Norwegian Women and Cancer study (NOWAC) [15,16], population-based studies on whole blood gene expression in postmenopausal women are scarce. However, some research on blood cells or tissue biopsies has reported gene expression patterns associated with HT and other menopause-related variables [17-22].

The population-based NOWAC postgenome cohort study provides opportunities for conducting nested case-control studies using gene expression analyses of whole blood [23]. A first step would be to assess the pre-disease impact of known risk factors for female cancer (e.g., circulating sex hormone levels, or HT) on gene expression.

The objective of this study was to explore potential associations between different levels of endogenous and exogenous sex hormones and gene expression in whole blood from a random sample of postmenopausal women.

## Methods

### Subjects

An extensive description of NOWAC has been published elsewhere [24]. Briefly, NOWAC is a national, population-based cohort study among women 30-70 years old, with questionnaire data on lifestyle and health collected at 4-6 year intervals.

**Figure 1:**
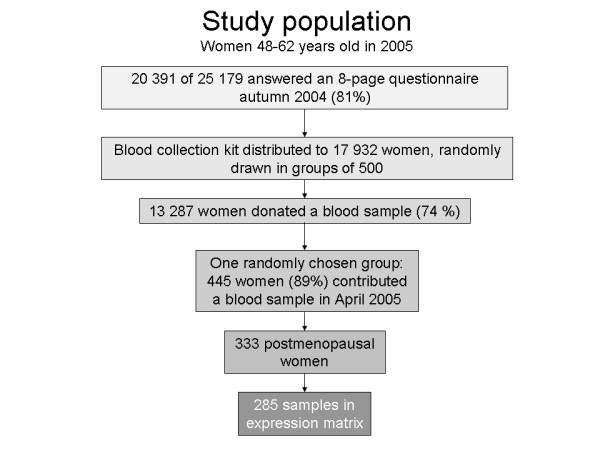
**Study population**.

The participants were randomly drawn from the Norwegian Central Population Register. By June 2007, approximately 172 000 women were enrolled in NOWAC overall. The study complied with the Declaration of Helsinki and all participants gave written informed consent. The study was approved by the Regional Committee for Medical and Health Research Ethics and the Norwegian Data Inspectorate. The NOWAC postgenome cohort comprises questionnaire data and blood samples collected during the 2003-2006 period from approximately 50 000 women born from 1943 to 1957 [23]. See Additional file 1 for a copy of the questionnaire (translated). For the present analyses, we used a randomly drawn group of 445 third-time participants from the NOWAC postgenome cohort who donated a blood sample in 2005 (Figure 1). The overall response rate was 74%. The exclusion of subjects with incompletely filled blood collection tubes, >3 days from blood collection to frozen sample or pre-/perimenopausal status, left 328 PAXgene (PreAnalytiX GmbH, Hembrechtikon, Switzerland) whole blood samples for RNA extraction.

### Endogenous hormones measurements

Plasma concentrations of estradiol (E_2_), progesterone (P_4_), testosterone (T), follicle stimulating hormone (FSH) and sex hormone binding globulin (SHBG) were measured by immunometry, as described in a previous report[7]. For convenience, SHBG is referred to as a hormone throughout this text.

### RNA isolation

Total RNA was isolated using the PAXgene Blood RNA Isolation Kit, according to the manufacturer's protocol (PreAnalytiX GmbH, Hembrechtikon, Switzerland). The RNA quantity and purity were assessed by the NanoDrop ND-1000 spectrophotometer (ThermoFisher Scientific, Wilmington, Delaware, USA). The absorbance ratio for 260 nm and 280 nm (A260/A280) was between 1.93 and 2.1 for all of the samples included for further analysis. The Experion automated electrophoresis system (BioRad, Hercules, CA, USA) and the RNA StdSens Analysis Kit were used to evaluate the RNA integrity of a randomised 32% of the samples, according to the manufacturer's protocol. Thirty-nine samples were excluded because of insufficient purity or yield.

### Microarray-based profiling and image analysis

The samples were analysed using the Applied Biosystems expression array system (Foster City, LA, USA). From each sample, 500 ng of total RNA was amplified using the NanoAmp RT-IVT labelling kit for one round of amplification (Applied Biosystems), in accordance with the manufacturer's protocol. The quantity and purity of the cRNA was measured using the NanoDrop ND-1000. Digoxigenin (DIG)-labelled cRNA, 10 μg per sample, was fragmented and hybridised to the Applied Biosystems Human Genome Survey Microarray V2.0, in accordance with the Chemiluminescence Detection Kit Protocol. Each microarray chip contained 277 control probes and 32 878 probes representing 29 098 genes. The Applied Biosystems Expression System software was used to extract signal intensities, signal to noise ratios (S/N) and flagging values from the microarray images.

### Data analysis

The data were analysed using R version 2.8.1 (http://cran.r-project.org) and tools from the Bioconductor project (http://www.bioconductor.org). For genes with a flagging value >8191, the expression intensity was set to missing. Three samples where less than 40% of the probes showed S/N ≥3 were excluded. Probes with S/N ≥3 in less than 50% of the samples were filtered out. We subsequently performed log transformation, quantile normalisation, and imputation of missing values using the k-nearest neighbourhood method (k = 10). The gene expression values were adjusted for significant technical variables (i.e., array lot number, RNA extraction date and time between blood collection and storage) using gene-wise mixed linear modelling [25]. One sample was excluded as an outlier because of high plasma E_2 _and P_4 _concentrations, which was probably due to misclassification of menopausal status. The final expression matrix comprised 285 samples and 16 185 probes. The microarray data have been deposited at Gene Expression Omnibus (GEO; http://www.ncbi.nlm.nih.gov/geo), accession number GSE15289.

The effect of different HT regimens and hormone concentrations on the expression of individual genes was tested using a linear model, limma[26], adjusted for multiple testing using the false discovery rate (FDR) [27].

The gene sets defined from the limma analyses, which were extracted from the literature or found in publicly available web applications, such as KEGG (Kyoto Encyclopaedia of Genes and Genomes) [28] or AmiGO [29], were tested for differential expression between groups with high and low hormone concentration using globaltest [30]. The table in Additional File 2 lists all the 56 gene sets included in the analysis. The gene sets fulfilling the criteria p < 0.05, FDR <0.25, and comparative p < 0.20 were defined as differentially expressed. The comparative p-value denotes the percentage of random gene sets of the same size that would have a larger test statistic than the gene set in question. The same method was used to test for gene set enrichment between the different categories of HT use versus non-use. Core genes for each significant gene set were defined as the genes with the highest influence on the differences seen (with a cut-off of z.score >1.5).

The analyses were adjusted for the variables that were significantly different between the groups in question (i.e., age and/or body mass index (BMI)).

Information on specific genes was found at GeneCards^® ^(http://www.genecards.org) [31]. Some genes were not assigned an approved gene symbol and are referred to as "unassigned" or by the Celera Gene ID, if one was provided in the Applied Biosystems annotation.

The searches for gene networks and pathways were performed using DAVID (the Database for Annotation, Visualisation and Integrated Discovery) [32] and HEFalMp (Human Experimental/Functional Mapper) [33].

Endogenous hormones were analysed as dichotomised variables: high (fourth quartile) versus low (first quartile) of hormone concentration, with cut-offs defined among the non-users of medication. Users of HT and thyroxine (T_4_) were excluded from these analyses.

Exogenous hormones were categorised as use of systemic E_2 _or E_2 _and progestogen (P) (tablets or patches), systemic E_2 _alone (patches), tibolone or total HT. Although not defined as postmenopausal HT, T_4 _use was also defined as a category. The different types of exogenous hormones were compared to non-use. Users of other medication (e.g., blood pressure lowering agents, antibiotics, antihistamines) were excluded from these analyses.

## Results

### HT use

Among the 285 women, 182 were medication users (52 used HT and 159 used other medication). Table 1 shows the participant characteristics for the study sample.

**Table 1:** Participant characteristics given as mean (SD) or frequency (%)

Age, years	55.7 (3.6)		
BMI, kg/m^2^	25.6 (4.3)		
		Among women not using medication	
Sex hormone concentration		1.quartile (low) Cut-off (mean)	4.quartile (high) Cut-off (mean)
Estradiol nmol/L	0.10 (0.09)	<0.05] (0.05)	>0.08 (0.14)
Progesterone nmol/L	0.99 (0.67)	<0.55] (0.44)	>1.21 (1.79)
Testosterone nmol/L	1.16 (0.74)	<0.66] (0.50)	>1.54 (2.14)
FSH IU/L	70.2 (28.0)	<56.4] (44.5)	>91.2 (106.4)
SHBG nmol/L	47.6 (21.8)	<32.0] (24.2)	>61.0 (75.1)
Medication use	182 (64%)		
HT	52 (18%)		
E_2 _and E_2_/P systemic	32 (62%)		
E_2 _systemic alone	9 (17%)		
Tibolone	10 (19%)		
Vaginal treatment	9 (17%)		
Thyroxine	20 (7%)		
Other medication	159 (56%)		
No medication	98 (34%)		
Number of medications used (n = 182)			
1 medication	97 (53%)		
2 medications	62 (34%)		
>2 medications	23 (13%)		
Current smoker			
Yes	75 (26%)		
No	209 (73%)		

**Figure 2:**
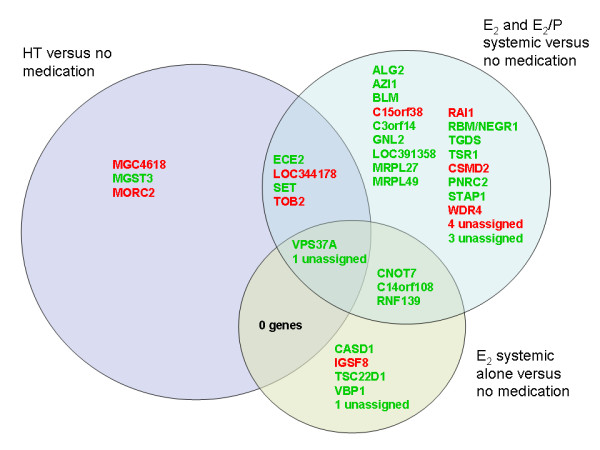
**The three gene sets (circles) found when comparing different categories of HT users with non-users**. The partly overlapping circles show which genes were significant for one, two or all three HT categories. All three categories include users of systemic E_2_. Gene symbols in red denote genes up-regulated in users and gene symbols in green denote genes up-regulated in non-users. Among the 9 HT genes, *LOC344178, SET, MGC4618 *and *MORC2 *have FDR <0.28, the remaining 5 genes have FDR <0.25.

Table 2 shows the results from the limma analysis, comparing different categories of HT with non-users (users of other medication excluded). Five genes were significantly associated with HT use, and nine genes with FDR <0.28. The list of differentially expressed genes associated with use of "E_2 _or E_2_/P systemic" treatment was the longest (n = 33). The overlap between the gene sets from the three HT categories that contain estradiol is shown in Figure 2. Tibolone and T_4 _use resulted in 400 and 8 differentially expressed genes, respectively. Because of the limited number of tibolone-only users (n = 2), a complementary analysis comprising all tibolone users was conducted. With an FDR cut-off at 0.50, we found 58 genes; no genes had FDR <0.41. Among these 58 genes, 21 were significantly associated with the tibolone-only users.

**Table 2:** Number of genes differentially expressed between HT users and non-users (in the Limma analysis)

HT type	Total sample N	Single users^1 ^N	Number of genes FDR <0.25
HT all types	52	23	5(9)^2^
E_2 _or E_2_/P systemic	32	15	33
E_2 _systemic	9	7	10
Tibolone	10	2	400
Tibolone2^3^	10	10	0(58)^3^
Thyroxine	20	5	8
Non-users	98	98	

^1 ^Users of other medication excluded.

^2 ^FDR <0.28, 5 genes with FDR <0.25

^3 ^All tibolone users were included in the analysis; there were 58 genes with FDR <0.50

**Figure 3:**
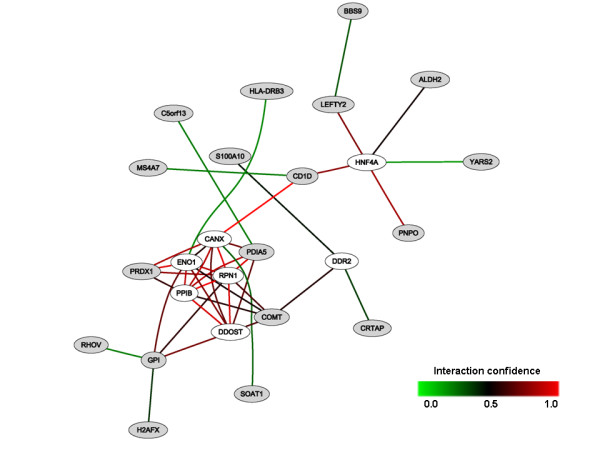
**The tibolone gene set (21 genes) in HEFalMp**. The network between core genes (in grey) related to tibolone use and genes (in white) predicted by Hefalmp in relation to this query, considering all genes in all biological processes.

Using functional annotation clustering in DAVID, the "E_2 _or E_2_/P systemic" gene set was enriched (enrichment score 1.67) in one cluster that included six general cellular component GO terms. Among them was "intracellular membrane bound organelle", which had the lowest FDR (0.17). The "E_2 _alone" gene set and the T_4 _gene set revealed no enriched clusters. HEFalMp predicted a few gene networks for the tibolone gene set (Figure 3). The tibolone gene set revealed no significantly enriched annotation clusters in DAVID. However, one individual GO term, "alcohol metabolic process" which was based on four genes (ALDH2, PRDX1, PDIA and PNPO), was significant (FDR = 0.06).

### High and low hormone concentrations

Comparing the high and low hormone concentrations using a gene-wise approach (limma, HT and T_4 _users excluded), two genes of unknown biological function (*DGCR9 *and *hCG2018460*) were significantly differentially expressed between high and low levels of FSH. No genes were significant for any of the remaining hormones. However, we observed significant gene set enrichment between high and low concentrations of E_2 _and P_4 _(the table in Additional file 3, with HT and T_4 _users excluded).

Out of the 22 gene sets significant for E_2_, seven were oestrogen or HT related, including the "E_2 _or E_2_/P systemic" gene set. Seven gene sets extracted from four publications were related to white blood cells and immune response, and the remaining eight (from five publications) were related to proto-oncogenes, exercise, age, consuming a carbohydrate/protein breakfast, transcription factors and drug metabolising enzymes. Among the 15 gene sets differentially expressed for P_4_, 11 were also significant for E_2_, although with slight differences in the core genes. Among the 186 different core genes, 151 (81%) were up-regulated in women with low E_2_/P_4 _concentrations. Among these, 71 (47%) were present for E_2_, 34 (23%) were present for P_4 _and 45 (30%) appeared on both lists. Among the 35 core genes up-regulated in women with high E_2_/P_4 _concentrations, 19 (54%) genes were present for E_2_, 14 (40%) were present for P_4 _and 2 (6%) were on both lists. When investigating FSH, SHBG and T, no gene set fulfilled all three criteria for differential expression in the gene set analysis. Of note, the same analysis conducted without excluding HT and thyroxine users only marginally altered the ranking of the gene sets by p-value, but the p-values and FDRs increased slightly (data not shown).

**Figure 4:**
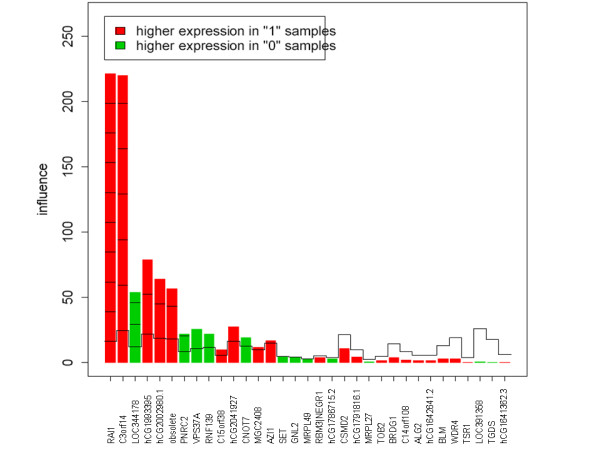
**Gene plot of the "E_2 _or E_2_/P systemic" gene set (33 genes) in relation to E_2 _concentration ("0" = low, "1" = high)**. The reference line for each bar represents the expected height under the null hypothesis (i.e., that the gene is not associated with hormone concentrations). The marks indicate the number of standard deviations above the reference line.

Our "E_2 _or E_2_/P systemic" gene set was only significant for E_2_, and therefore it represents a novel oestrogen gene expression signature. Among the six core genes for the "E_2 _or E_2_/P systemic" gene set (Figure 4), five had unknown function, while *RAI1 *may function as a transcription regulator. Comparing the direction of gene expression (up- or down-regulation) for the "E_2 _or E_2_/P systemic" gene set between endogenous and exogenous E_2 _(Figures 2 and 4), we found 61% concordance for all 33 probes and 80% concordance among the 10 most influential probes. The second most influential gene (*C3orf14*) was up-regulated in high E_2 _but down-regulated among users of systemic E_2 _relative to non-users. Gene number three (*LOC344178*) was down-regulated in high E_2 _but up-regulated among users of systemic E_2_. None of the five genes that overlapped between the "E_2 _alone" and "E_2 _or E_2_/P systemic" gene sets (Figure 2) were among the six core genes; they were ranked as numbers 8, 9, 12 and 21 (Figure 4).

## Discussion

This study confirms that a population based cohort study such as NOWAC provides the opportunity to use high throughput technology (e.g., microarray analysis) to explore biologic variation in gene expression related to both endogenous and exogenous sex hormones.

According to the gene-wise analysis, hormone concentrations did not show a profound influence on gene expression. This result is not surprising, given the low variability that is present in a study group representing the general postmenopausal population. Conversely, all categories of HT use produced differentially expressed genes when compared with non-users. This finding is attributable to the wider range of hormone concentrations between the groups in this analysis. Intake of exogenous E_2_, particularly by systemic administration, increases endogenous plasma E_2 _and suppresses plasma FSH toward premenopausal levels [7]. Apart from the direct hormonal effects, a probable cause is the supply of synthetic medical substances (e.g., tibolone, progestogens and their metabolites) to the blood. The overlap between the gene sets shown in Figure 2 was probably caused by the overlap of subjects; all of the women using systemic E_2 _alone are included among the women using E_2_P systemic, who are again included among the HT-users. Also, these genes seem the most stable, as they remain significant even if the sample group composition is changed. The minimal information on gene function for the two E_2 _gene sets in DAVID could be due to the nature of gene-wise analysis, which assumes that genes are expressed independently of each other.

The gene set enrichment analysis showed a fair amount of overlap between P_4 _and E_2_, a plausible result considering the positive correlation between the two hormones (r = 0.43, p < 0.01). Among the 58 subjects present in both the E_2 _and the P_4 _analyses, 49 were concordantly in the low or high group for both hormones. Hence, it may be difficult to disentangle gene expression associated with E_2 _and P_4_. Still, there are some differences. For example, the "oestrogen up-regulated" gene set (Frasor/KEGG) was only significant for E_2_, and although the total "oestrogen regulated" gene set (Frasor/KEGG) was significant for both E_2 _and P_4_, there was no overlap between the core genes up-regulated in the high group. In general, we found a much larger overlap of core genes up-regulated in the low than in the high group for the gene sets that were significant for both hormones.

The "E_2 _or E_2_/P systemic" gene set turned out to be a more reliable oestrogen signature than the "E_2 _alone" gene set, probably because of the inclusion of oral high-dose E_2 _users (n = 7) and/or the generally larger group of users in the "E_2 _or E_2_/P systemic" category. Interestingly, as opposed to most of the other significant gene sets, the majority of the genes in this gene set were up-regulated in the high E_2 _group. There was a high, although not complete, concordance in the direction of gene expression between endogenous and exogenous E_2 _for this gene set. Opposing directions for some genes may have been due to the progestogen content in several of the products in this HT category, or possibly differential feedback mechanisms between endogenous and exogenous hormones. Further research may reveal the functions and regulation of these core genes.

In addition to the core genes, interesting single genes in the "E_2 _or E_2_/P systemic" gene set included *PNRC2*, a coactivator of nuclear receptors such as the *ESRR*s (oestrogen related receptors), and *CSMD2*, which has been previously found to be differentially expressed in HT users [15]. Among the remaining genes aspiring to, but not quite reaching, core gene status (Figure 4), are also *VPS37A *(no. 8), *RNF139 *(No. 9) and *CNOT7 *(No. 12), indicating that these overlapping genes from Figure 2 are worthy of further research into their association with sex hormones. Noteworthy in the "E_2 _alone" gene set is the *TSC22D1 gene*, whose protein product may play a role in resistance toward Tamoxifen^® ^treatment in breast cancer patients[34]

The tibolone and thyroxine gene sets did not meet our significance criteria for any of the hormones. One might have expected some association with FSH, but the number of users in these two categories was probably too small to generate reliably specific expression sets.

None of the gene sets were differentially expressed between high and low levels of FSH, SHBG or T. Compared with the wide variety of target tissues and the acknowledged effects of steroid hormones, FSH and SHBG would be expected to have a more limited association with gene expression. The biological effect of FSH is essentially the stimulation of gonadal E_2 _and P_4 _synthesis, and in postmenopausal women FSH has lost its gonadotropic potency. Although it has been suggested that SHBG possesses some signalling properties [35], it is mainly a transport protein. Adding the moderate variation in FSH and SHBG levels across the study population, a difference in gene expression might be difficult to detect. Testosterone is not a major hormone in women. Although it is a potent steroid, the differences in gene expression relative to low levels of T are probably not detectable in a setting with high background variability.

Seven gene sets related to immune responses or cells active in the immune system were differentially expressed between the high and low E_2 _concentration groups. Additionally, two gene sets associated with exercise (stress response and inflammatory response) and the proto-oncogene gene set could be viewed as immune system related. Sex hormones have been found to influence the immune system through steroid receptors in white blood cells [36]. In general, female sex hormones are viewed as suppressors of the immune response. It has been shown that plasma levels of both interleukin 6 (IL6) and interleukin 2 (IL2) increase after menopause (i.e., with decreasing levels of E_2_) and that HT opposes this effect [36]. Although neither IL6 nor IL2 were among the 16 185 probes in our data set, the higher expression of the respective receptors, *IL6R *and *IL2R*, at low E_2 _concentrations indicates the suppressive effects of E_2_. Other interesting core genes include the heat shock proteins (*HSP*s) in the "stress response from exercise" gene set. The *HSP*s function as intracellular chaperones for other proteins (integrity and folding), and some have been found to play a role in the rapid non-genomic effects of steroid hormones [37], which is interesting in light of the rapid responses seen in these genes following exercise [38]. *FOS *is a high-influence core gene for both E_2 _and P_4_. In fact, all of the *FOS*-containing gene sets were differentially expressed. However, contradictory to Frasor et.al. [39], *FOS *was up-regulated in the low E_2 _group, together with *EPB41L3 *and *AP1G1*. By contrast, *CXCL12*, the steroid 21-hydroxylase *CYP21A2 *and *PDZK1 *were congruously up-regulated in the high-E_2 _group. According to Kendall et al. [20], *FOS *is up-regulated by oestrogen deprivation, which supports our results, while *SGK3 *and *TAGLN *are down-regulated, which contradicts our results. These contradictions in gene expression direction may arise from methodological differences or from regulatory and feed-back mechanisms similar to the above mentioned discordance for the "E_2 _or E_2_/P systemic" gene set.

Though the differential expression of the tibolone gene set lacked statistical significance, the network mapping suggested that further research is warranted. Interesting single genes included *COMT*, a central enzyme in the metabolism of oestrogens; and *SOAT1*, an intracellular protein that forms cholesterol esters from cholesterol, thereby possibly contributing to atherosclerotic plaques. *SOAT1 *was up-regulated among tibolone users, in accord with the known increased risk of stroke associated with tibolone use [40]. A larger data set would contain a larger group of tibolone users and provide a more solid basis for finding tibolone associated genes.

### Strengths and limitations

The NOWAC study subjects were randomly drawn from the Central Population Register and are representative of the population in which future microarray based diagnostic and/or prognostic tests for breast cancer will be applied. Our ability to detect subtle effects in a dataset with a high degree of random variation is reassuring.

Among the limitations of this study is the lack of information regarding the relative proportions of peripheral blood cell types. If differences in hormone concentrations or HT use are associated with the numbers of particular type(s) of peripheral blood cells, it may have influenced our results. Research into the influence of sex hormones on leukocyte cell count reveals conflicting results [36]. Although the women were healthy enough to visit a physician's office, we had limited information regarding disease and immune system status beyond what could be inferred from the self-reported drug use. However, a systematic difference in disease prevalence between hormone concentration levels is unlikely.

Our FDR cut-off of <0.25 may have exceeded conventional limits, whereas FDR≤0.10 is considered acceptable [41]. A higher FDR can be accepted, however, at least when analysing gene sets extracted from previous publications and thereby supported by research. Also, we were not looking for single genes in the gene-wise analyses but for groups of genes that may have explained known effects. For example, among the 33 genes in the "E_2 _or E_2_/P systemic" gene set, 9 had FDR≤0.10, but only two of these genes were among the core genes differentially expressed between the high and low E_2 _group. Hence, if we had used the ≤0.10 FDR cut-off we might have overlooked this oestrogen signature.

Our results are based on a snapshot measurement; we have only one blood sample from each woman and can infer nothing about intra-individual variation or variation over time. However, previous reports have shown low intra-individual variation in gene expression compared with inter-individual variation [42,43].

The study design prevented an extensively standardised blood sampling protocol with regards to fasting, blood sample handling and transport, etc. However, the main source of technical variation in this data set was associated with the performance of the assay and not with pre-analytical processing[25].

The gene set enrichment analyses were adjusted for age and/or BMI. We found no significant differences between the compared categories with respect to fasting and smoking. However, residual confounding may have influenced the differences found between high and low concentrations of E_2 _and P_4_.

Differentially expressed genes have not been validated using an independent data set, and our results should be interpreted accordingly.

## Conclusions

Through exploring potential associations between sex hormones and gene expression, we have identified a novel oestrogen gene expression signature, and further research may reveal the function of these genes in relation to E_2_. A potential tibolone signature was also defined that warrants further research. Several gene sets, particularly those oestrogen and immune system related but also gene sets related to drug metabolism, exercise and carbohydrate/protein ingestion, were differentially expressed between high and low levels of E_2 _and P_4_.

## Competing interests

The authors declare that they have no competing interests.

## Authors' contributions

MW, VD and EL participated in designing the study. MW performed the statistical analyses and drafted the manuscript. KSO executed the laboratory microarray analyses. CR and VD assisted in statistical analyses and the drafting of the manuscript. EL is the principal investigator of the NOWAC study. All authors read and approved the final manuscript.

## Pre-publication history

The pre-publication history for this paper can be accessed here:

http://www.biomedcentral.com/1755-8794/4/29/prepub

## Supplementary Material

Additional file 1 **Questionnaire, translated from Norwegian**. This is a PDF-file of the questionnaire that the women completed at the time of the blood draw. Click here for file

Additional file 2 **Gene sets included in the gene set enrichment analysis**. This is a Word table showing all the 56 gene sets included in the analysis. The gene sets are categorised as related to female sex hormone or menopause, as related to blood cells and the immune system, or, as related to other factors. Click here for file

Additional file 3 **Gene sets differentially expressed between high and low hormone concentrations, age adjusted with HT and thyroxin users excluded**. This is a Word table showing which of the 56 gene sets that showed a statistically significant differential expression between the women with high and low plasma concentrations of estradiol and progesterone. Click here for file
