# Supplementary material for: Sex hormones and gene expression signatures in peripheral blood from postmenopausal women - the NOWAC postgenome study
Source: BMC Med Genomics. 2011 Mar 31;4:29. doi: 10.1186/1755-8794-4-29 (PMC3078834; doi:10.1186/1755-8794-4-29)
Supplement: Additional file 1 — Questionnaire, translated from Norwegian. This is a PDF-file of the questionnaire that the women completed at the time of the blood draw. [file 1755-8794-4-29-S1.PDF]

NOWAC questionnaire that accompanies the blood samples

2005
CONFIDENCIAL

The questionnaire must be answered in connection with the blood draw.

ID:

The questionnaire MUST accompany the blood sample

LAB:

I have read the information concerning the blood sample donation and I concent to participate:

Yes ☐

Blood draw

When was the blood sample drawn?

Date (day, month) ☐☐ ☐☐
Time (hour,minute) ☐☐ ☐☐

When was your latest meal before blood draw?

Date (day, month) ☐☐ ☐☐
Time (hour,minute) ☐☐ ☐☐

Posture during blood draw:

Sitting ☐
Laying down ☐

Menstruation

Do you have menstruations?

Yes ☐
No ☐
Irregular ☐
Pregnant ☐

If yes, please provide the date for the first day of your last menstruation:
(day, month) ☐☐ ☐☐

Smoking during the past week

Have you smoked during the past week?

Yes ☐
No ☐

If yes, how many cigarettes did you smoke

Yesterday ☐
Today ☐

Weight/height

What do you weigh today? kg ☐☐☐
How tall are you? cm ☐☐☐

Were weight and height measured at the doctor's office today?

Yes ☐
No ☐

Medication during the past week

Have you used oral contraceptives during the past week?
Yes ☐
No ☐

If yes, please provide the date for the last tablet taken: (day, month) ☐☐ ☐☐

Have you used hormone tablets/patches (estrogen, gestagen) for climacteric complaints during the past week?
Yes ☐
No ☐

If yes, please provide the date when the last tablet was taken:
(day, month) ☐☐ ☐☐

Product name: ______________
Product name: ______________

Have you used any other medication during the past week?

Yes ☐
No ☐

If yes, please provide the date when the medication was last taken:

(day, month) ☐☐ ☐☐

Product name: ______________

(day, month) ☐☐ ☐☐

Product name: ______________

(day, month) ☐☐ ☐☐

Product name: ______________

Dietary supplements use during the past week

Have you taken cod liver oil (liquid) during the past week?
Yes ☐
No ☐

If yes, please provide the date for the last dose (day, month) ☐☐ ☐☐

How much did you take at that time?
1 teaspoon ☐
1/2 tablespoon ☐
≥ 1 tablespoon ☐

Have you taken capsules containing cod liver oil/omega-3/fish oil during the past week?
Yes ☐
No ☐

If yes, please provide the date for the last dose (day, month) ☐☐ ☐☐

How many capsules did you take at that time?
1 ☐
2 ☐
≥ 3 ☐

Product name: ______________

Have you taken soy supplements during the past week?
Yes ☐
No ☐

Product name: ______________
Product name: ______________

Have you taken any other dietary supplements (vitamins/minerals) during the past week?
Yes ☐
No ☐

If yes, please provide the date for the last dose (day, month) ☐☐ ☐☐

Product name: ______________
Product name: ______________
