# Supplementary material for: Sex hormones and gene expression signatures in peripheral blood from postmenopausal women - the NOWAC postgenome study
Source: BMC Med Genomics. 2011 Mar 31;4:29. doi: 10.1186/1755-8794-4-29 (PMC3078834; doi:10.1186/1755-8794-4-29)
Supplement: Additional file 2 — Gene sets included in the gene set enrichment analysis. This is a Word table showing all the 56 gene sets included in the analysis. The gene sets are categorised as related to female sex hormone or menopause, as related to blood cells and the immune system, or, as related to other factors. [file 1755-8794-4-29-S2.DOC]

#### Additional file 2 Gene sets included in the gene set enrichment analysis

| **Reference** | **RNA source** | **Expression profiling method** | **Gene sets** | **genes N** | **probes N** | **Subjects N/sex*** |
| --- | --- | --- | --- | --- | --- | --- |
| **Gene sets related to female sex hormones or menopause** | | | | | | |
| Present study | Whole blood | Applied Biosystems | Genes associated with hormone therapy use | 9 | 9 | 121w |
|  |  | Applied Biosystems | Genes associated with systemic E_2_ or E_2_/P use | 33 | 33 | 113w |
|  |  | Applied Biosystems | Genes associated with systemic E_2_ use | 10 | 10 | 105w |
|  |  | Applied Biosystems | Genes associated with tibolone use alone | 347 | 400 | 100w |
|  |  | Applied Biosystems | Genes associated with tibolone use, all users | 54 | 58 | 108w |
|  |  | Applied Biosystems | Genes associated with tibolone use combined | 21 | 21 | - |
|  |  | Applied Biosystems | Genes associated with thyroxine use | 8 | 8 | 103w |
| AmiGO [1] |  |  | Response to oestrogen stimulus, GO:0043627. GO database release 2009-09-17 | 23 | 27 | - |
| Frasor [2] | MCF-7 (breast cancer cell line) | Affymetrix Hu95A GeneChips | Oestrogen up-regulated genes (incl. 28 hormone related genes from KEGG (ref)) | 56 | 68 | - |
|  |  |  | Oestrogen regulated genes (incl. 28 hormone related genes from KEGG (ref)) | 134 | 174 | - |
| Dumeaux [3] | Whole blood | Agilent 22k oligoarray | Genes predicting hormone therapy use | 83 | 112 | 100w |
| Ji[4] | MCV152 (ovarian surface epithelium) | Affymetrix Human Genome HGU95Av2 GeneChips, 10,000 full-length genes. | FSH treatment at 200 mIU/mL for 72 hr or no treatment. (Differential exprs. defined by fold change) | 113 | 142 | 26w |
| Kendall[5] | Breast biopsies | in-house Breakthrough cDNA microarrays, two color, dye swap | Normal and cancer biopsies before and after Letrozol treatment (oestrogen deprivation) | 45 | 57 | 13w |
| Dumeaux[6] | Whole blood | Applied Biosystems | Genes associated with hormone therapy use, core genes, 3 methods for globin RNA reduction | 7 | 10 | 12w |
|  |  |  | Genes associated with hormone therapy use after globin reduction using peptide nucleic acids (PNA) | 14 | 19 | 12w |
|  |  |  | Genes associated with hormone therapy use, no globin reduction | 11 | 14 | 12w |
| Pöllänen[7] | Muscle biopsies | Sentrix HumanRef-8 Expression BeadChips (BD-25-201, Illumina) | Hormone therapy use vs. non-use | 35 | 50 | 15w |
| Dvornyk[8] | Monocytes | Affymetrix GeneChip Human U133A | Pre- vs. postmenopausal women | 40 | 57 | 19w |
| Xiao[9] | B-cells | Affymetrix HG-U133A GeneChip | Low vs. high Bone Mineral Density (BMD) | 29 | 34 | 20w |
| KEGG[10] |  |  | Oestrogen-androgen metabolism | 18 | 20 | - |
|  |  |  | Steroid hormone metabolism | 10 | 10 | - |
| **Gene sets related to blood cells and the immune system** | | | | | | |
| KEGG[10] |  |  | T cell receptor signalling | 94 | 110 | - |
| Tanner[11] | Whole blood | 5' Nuclease PCR assay | Interleukins | 15 | 17 | 1m |
|  |  |  | General cytokines | 11 | 11 | 1m |
|  |  |  | CD markers | 20 | 23 | 1m |
| Whitney[12] | PBMC and whole blood | Standford cDNA microarray | Lymphocyte signature | 55 | 76 | 35w/40m |
|  |  |  | Monocyte signature | 25 | 29 | 35w/40m |
|  |  |  | PBMC signature | 78 | 105 | 35w/40m |
|  |  |  | Neutrophil signature | 31 | 38 | 35w/40m |
|  |  |  | Red blood cell signature | 37 | 44 | 35w/40m |
|  |  |  | Reticulocyte signature | 9 | 7 | 35w/40m |
|  |  |  | Red blood cell and reticulocyte signature | 48 | 51 | 35w/40m |
| Martinelli[13] | Neutrophils | Affymetrix HG-U95Av2 GeneChip | Immature vs. mature neutrophils | 25 | 29 | - |
| Cobb[14] | Whole blood | Affymetrix U133A or U133 Plus GeneChip | T cell enriched vs. leukocytes | 104 | 121 | 5 |
|  |  |  | Monocyte enriched vs.leukocytes | 83 | 102 | 5 |
|  |  |  | Up-regulated genes associated with trauma | 77 | 89 | 31 |
|  |  |  | Down-regulated genes associated with trauma | 103 | 139 | 31 |
| Other |  |  | Immunoglobulin gene set | 51 | 51 | - |
| Eady[15] | PBMC | In house oligo-microarray | Natural killer cells in PBMC signature | 26 | 35 | 10w/6m |
|  |  |  | Monocytes in PBMC signature | 47 | 61 | 10w/6m |
| **Gene sets related to other factors** | | | | | | |
| Eady[15] | PBMC | In house oligomicroarray | Genes related to age | 15 | 15 | 10w/6m |
|  |  |  | BMI-specific genes | 3 | 3 | 10w/6m |
| Tanner[11] | Whole blood | 5'Nuclease PCR assay | Proto-oncogenes | 7 | 8 | 1m |
| Connolly[16] | PBMC | Affymetrix HU133A GeneChip. | Stress response associated with exercise | 12 | 15 | 15m |
|  |  |  | Growth factor and transcription factor associated to exercise | 23 | 27 | 15m |
|  |  |  | Inflammatory response to exercise | 24 | 27 | 15m |
| Radich[17] | Leukocytes | Agilent Hu25K microarray | Individual specific genes | 50 | 66 | 8w/7m |
| Lampe[18] | Whole blood | Agilent Hu25k microarray | Genes correlated with cotinine levels | 28 | 43 | 37w/48m |
|  |  |  | Positive correlation with cotinine levels | 20 | 30 | 37w/48m |
|  |  |  | Negative correlation with cotinine levels | 8 | 13 | 37w/48m |
| van Erk[19] | Leukocytes | Affymetrix U133A GeneChip | Genes associated with high protein high carbohydrate breakfasts | 27 | 33 | 8m |
|  |  |  | Protein synthesis genes associated to high protein breakfast | 32 | 73 | 8m |
|  |  |  | High inter-individual variability genes | 20 | 28 | 8m |
| Siest[20] | Lymphocytes | In-house microarray (Visvikis-Siest et al., 2007) | Drug metabolizing enzymes | 16 | 23 | 9w/11m |
|  |  |  | Transcription factors | 12 | 16 | 9w/11m |
|  |  |  | Transcription factors and drug metabolizing enzymes | 28 | 39 | 9w/11m |
| * w=women, m=men | | | | | | |

**References**
