# Supplementary material for: Sex hormones and gene expression signatures in peripheral blood from postmenopausal women - the NOWAC postgenome study
Source: BMC Med Genomics. 2011 Mar 31;4:29. doi: 10.1186/1755-8794-4-29 (PMC3078834; doi:10.1186/1755-8794-4-29)
Supplement: Additional file 3 — Gene sets differentially expressed between high and low hormone concentrations, age adjusted with HT and thyroxin users excluded. This is a Word table showing which of the 56 gene sets that showed a statistically significant differential expression between the women with high and low plasma concentrations of estradiol and progesterone. [file 1755-8794-4-29-S3.DOC]

#### Additional file 1 Gene sets differentially expressed between low and high hormone concentration, age adjusted with HT and thyroxin users excluded

|  |  |  |  |  |  |  | **Core genes^§^ (probes) up-regulated in 1.quart.** | | **Core genes^§^ (probes) up-regulated in 4.quart.** | |
| --- | --- | --- | --- | --- | --- | --- | --- | --- | --- | --- |
|  | **Gene sets** | **N**  **total probes** | **N tested probes** | **p-value** | **FDR** | **Comp.**  **p-value** | **N** | **Gene symbols** | **N** | **Gene symbols** |
| **Estradiol (N=99)** | |  |  |  |  |  |  |  |  |  |
|  | Stress response from exercise[1] | 15 | 12 | 0,004 | 0,08 | 0,01 | 5(4) | *DUSP5, HSPA1A/HSPA1B, HSPH1, HSPCA* | 0 |  |
|  | T-cell receptor signaling, KEGG[2] | 114 | 72 | 0,007 | 0,08 | 0,00 | 27(26) | *FOS, RELA, NFKB1, RHOA, CD3Z, LCP2, CD3E, CHP, NFATC1, NFATC3, PIK3CG, MAP3K8, PIK3R5, PAK1, CDK4, FYN, ZAP70, MAP3K14, PPP3CA, CARD11, CD40LG, GRB2, CBL, LCK, LAT/SPIN1, PIK3CD* | 2 | *HRAS, CD8B1* |
| * | Oestrogen responsive genes (GO:0043627)[3] | 27 | 16 | 0,008 | 0,08 | 0,02 | 3(5) | *STAT3, CRIPAK, TGFB1* | 1 | *RNF14* |
|  | Neutrophil signature[4] | 38 | 31 | 0,011 | 0,08 | 0,01 | 11 | *IL6R, SIGLEC5, SLA, ZFP276, FPRL1, FPR1, CSF3R, GBP1, LYN, PSCDBP, PIK3CD* | 1 | *FANCA* |
| * | Predictors of HT use[5] | 112 | 52 | 0,011 | 0,08 | 0,02 | 9(10) | *PILRA, ACTC, TLE4, HLA-DQA1, GNLY, RNF24, IRF2, AVIL, GNAZ* | 3 | *C8B, GPR116, MALAT1* |
| * | E_2_ or E_2_/P systemic | 33 | 33 | 0,014 | 0,08 | 0,02 | 1 | *LOC344178* | 5 | *RAI1, C3orf14, 3 unassigned (hCG1993395, hCG2002980.1,*  *one obsolete)* |
|  | High carbohydr. and protein breakfast[6] | 33 | 29 | 0,017 | 0,08 | 0,04 | 6 | *SIGLEC5, DAPK1, PDCD4, C1QR1, KLRF1, DHRS9* | 0 |  |
|  | Monocytes in PBMC signature[7] | 61 | 50 | 0,017 | 0,08 | 0,03 | 10 | *APLP2, ATP6V1B2, CDA, ADRBK2, BRI3, CCND2, SERPING1, NRGN, LCK, FES* | 3 | *RNASE3, RIRPB1, PTPNS1* |
|  | Transcription factors and drug metabolizing enzymes[8] | 39 | 23 | 0,018 | 0,08 | 0,03 | 3 | *NR1H2, CYP4F2, TCF7* | 1 | *GSTT1* |
| * | Oestrogen related, Frasor/KEGG, up-regulated[2, 9] | 68 | 23 | 0,019 | 0,08 | 0,05 | 3(4) | *FOS, EPB41L3, AP1G1* | 3 | *CXCL12, CYP21A2, PDZK1* |
|  | Age[7] | 15 | 9 | 0,019 | 0,08 | 0,05 | 3 | *NEDD9, CHIC2, UTF1* | 1 | *HLA-DQB1* |
|  | Natural killer cells in PBMC sign.[7] | 35 | 25 | 0,020 | 0,08 | 0,05 | 7 | *CNOT2, KIR2DL4, CTBP2, MLC1, CX3CR1, KLRF1, CTSW* | 1 | *CD8B1* |
|  | Proto-oncogenes[10] | 8 | 6 | 0,022 | 0,08 | 0,07 | 2 | *FOS, NFKB1* | 0 |  |
|  | Drug metabolizing enzymes[8] | 23 | 12 | 0,022 | 0,08 | 0,04 | 1 | *CYP4F2* | 1 | *GSTT1* |
|  | PBMC signature[4] | 105 | 89 | 0,023 | 0,08 | 0,05 | 20(15) | *KIAA1219, GZMB, CSF1R, HLA-DRB1/HLA-DRB3, IL2RB, FAIM3, C1QR1, HLA-DQA1, GNLY, TRAJ17/TRDV2/TRAC/TRAV20/TRA@, PGD, TNFRSF7, CTSW, TRBV19/TRBC1* | 1 | *HLA-DQB1* |
|  | Trauma; down-regulated genes[11] | 138 | 119 | 0,023 | 0,08 | 0,03 | 26(22) | *LEF1, LBH, FAM102A, CD3E, RABGAP1L, IL2RB, FAIM3, P2RY10, SPOCK2, TP53, HLA-DQA1, HNRPA1, GNLY, TRAJ17/TRDV2/TRAC/TRAV20/TRA@, RPS4X, KLRF1, NOV, PRF1, LCK, TRBV19/TRBC1, RARRES3* | 1 | *TNFRSF25* |
| * | HT use, core genes, incl. PNA[12] | 19 | 12 | 0,024 | 0,08 | 0,06 | 3 | *LEF1, FOS, TLE4* | 1 | *GPR116* |
| * | Oestrogen related, Frasor/KEGG[2, 9] | 175 | 79 | 0,026 | 0,08 | 0,05 | 11(14) | *FOS, KYNU, EPB41L3, KIAA0922, ABCG1, AP1G1, LITAF, DBN1, GNE, KLF6, KRT7* | 3 | *CXCL12, CYP21A2, PDZK1* |
|  | Lymphocyte signature[4] | 73 | 61 | 0,029 | 0,09 | 0,06 | 16(12) | *KIAA1219, GZMB, CSF1R, IL2RB, FAIM3, HLA-DQA1, GNLY, TRAJ17/TRDV2/TRAC/TRAV20/TRA@. TNFRSF7, CTSW, TRBV19/TRBC1* | 0 |  |
| * | Response to oestrogen deprivation, breast tissue[13] | 57 | 18 | 0,032 | 0,09 | 0,12 | 3 | *FOS, SGK3, TAGLN* | 1 | *MALAT1* |
|  | Inflammatory response to exercise[1] | 27 | 25 | 0,038 | 0,10 | 0,12 | 8 | *IL6R, GZMB, IL2RB, NCR3, GNLY, CSF3R, PRF1, CTSW* | 0 |  |
|  | High interindividual variability genes[6] | 28 | 24 | 0,044 | 0,11 | 0,14 | 4(4) | *HLA-DRB1/HLA-DRB3, HLA-DQA1, IFIT2,* | 1 | *HLA-DQB1* |
| **Progesterone (N=104)** | |  |  |  |  |  |  |  |  |  |
| * | HT use, core genes, incl. PNA[12] | 19 | 12 | 0,005 | 0,14 | 0,010 | 4 | *LEF1, FOS, CREB5, TLE4* | 1 | *GPR116* |
|  | Interleukins[10] | 17 | 5 | 0,007 | 0,14 | 0,018 | 2 | *IL1B, IL15* | 1 | *IL7* |
|  | Monocytes in PBMC signature[7] | 61 | 50 | 0,008 | 0,14 | 0,005 | 16(18) | *SERPING1, BRI3, LMO2, CDA, ATP6V1B2, TIMP2, IGSF6, APLP2, CREB5, SERPINA1, FLJ20273, RAB31, PLSCR1, SLC31A2, BCL6, ADRBK2* | 1 | *RIN2* |
|  | Stress response from exercise[1] | 15 | 12 | 0,010 | 0,14 | 0,022 | 3(2) | *DUSP5, HSPA1A/HSPA1B* | 1 | *SPON2* |
|  | High carbohydr. and protein breakfast[6] | 33 | 29 | 0,016 | 0,14 | 0,030 | 8(8) | *SIGLEC5, DHRS9, PDCD4, PSAP, DAPK1, TNFSF13/TNFSF13-TNFSF12, HAL* | 0 |  |
| * | Oestrogen related, Frasor/KEGG[2, 9] | 175 | 79 | 0,019 | 0,14 | 0,041 | 14(14) | *LITAF, FOS, KYNU, IFI30, ABCG1, AP1G1, KIAA0922, DBN1, RAB31, ENC1, IER3, HIST2H2AA/HIST2H2AC, CBX6* | 5(6) | *RAP1GA1, TFF1, SELENBP1, ADCY9, AP1M2* |
|  | Neutrophil signature[4] | 38 | 31 | 0,022 | 0,14 | 0,048 | 9 | *GBP2, GBP1, IL6R, SIGLEC5, LYN, LILRA2, CSF3R, BCL6, SLA* | 0 |  |
|  | Growth factor, transcription factor, excercise[1] | 27 | 16 | 0,022 | 0,14 | 0,097 | 2 | *FOS, ECGF1* | 1 | *PDGFRB* |
|  | Proto-oncogenes[10] | 8 | 6 | 0,023 | 0,14 | 0,077 | 2 | *FOS, NFKB1* | 0 |  |
| * | Oestrogen responsive genes (GO:0043627) [3] | 27 | 16 | 0,025 | 0,14 | 0,104 | 2(4) | *STAT3, TGFB1* | 2 | *TFF1, GH1* |
|  | T-cell reseptor signalling, KEGG[2] | 114 | 72 | 0,028 | 0,14 | 0,114 | 20(23) | *PAK1, CHP, PPP3CA, FOS, NFKBIE, LCP2, NFATC1, CDC42, RELA, MAP3K14, LAT/SPIN1, CRB2, MAP3K8, CD40LG, PTPN6, AKT1, NFATC3, GRAP2, NFKB1* | 0 |  |
| * | Predictors of HT use[5] | 112 | 52 | 0,030 | 0,14 | 0,108 | 11(10) | *PILRA, RNF24, GNAZ, AVIL, SLC12A6, CREB5, TLE4, IRF2, HIST2H2AA/HIST2H2AC, QPCT* | 5 | *GPR116, GPHA2, C8B, GPR75, SLC36A1* |
| * | HT use, no globin reduction[12] | 14 | 11 | 0,039 | 0,17 | 0,130 | 3 | *FOS, CREB5, TLE4* | 0 |  |
| * | Response to oestrogen deprivation, breast tissue[13] | 57 | 18 | 0,048 | 0,17 | 0,179 | 2 | *FOS, TAGLN* | 1 | *IFT122* |
|  | General cytokines[10] | 11 | 5 | 0,050 | 0,17 | 0,156 | 3 | *LTB, TGFB1, FAS* | 0 |  |
| * Gene sets related to steroid hormones, ^§^ The core genes are listed according to z.score from highest to lowest (above 1.5). | | | | | | | | | | |

#### References
